# Electrochemical Shell-Isolated Nanoparticle-Enhanced Raman Spectroscopy of Imidazole Ring Functionalized Monolayer on Smooth Gold Electrode

**DOI:** 10.3390/molecules27196531

**Published:** 2022-10-03

**Authors:** Agnė Zdaniauskienė, Martynas Talaikis, Tatjana Charkova, Rita Sadzevičienė, Linas Labanauskas, Gediminas Niaura

**Affiliations:** 1 Center for Physical Sciences and Technology (FTMC), Department of Organic Chemistry, Sauletekio Ave. 3, LT-10257 Vilnius, Lithuania; 2 Life Sciences Center, Institute of Biochemistry, Department of Bioelectrochemistry and Biospectroscopy, Vilnius University, Sauletekio Ave. 7, LT-10257 Vilnius, Lithuania

**Keywords:** Ag@SiO_2_, core-shell nanoparticles, temperature-dependent Raman, hydrogen bonding interaction, histidine, SHINERS, RAIRS

## Abstract

The imidazole ring (Im) of histidine side chains plays a unique role in the function of proteins through covalent bonding with metal ions and hydrogen bonding interactions with adjusted biomolecules and water. At biological interfaces, these interactions are modified because of the presence of an electric field. Self-assembled monolayers (SAMs) with the functional Im group mimic the histidine side chain at electrified interfaces. In this study, we applied in-situ shell-isolated nanoparticle-enhanced Raman spectroscopy (SHINERS) to probe the structure and hydrogen bonding of Im-functionalized SAM on smooth Au at the electrochemical interface. The self-assembly of molecules on the Au induced the proton shift from N1 atom (Tautomer-I), which is the dominant form of Im in the bulk sample, to N3 atom (Tautomer-II). The impact of electrode potential on the hydrogen bonding interaction strength of the Im ring was identified by SHINERS. Temperature-Raman measurements and density functional theory (DFT) analysis revealed the spectral marker for Im ring packing (mode near 1496–1480 cm^−1^) that allowed us to associate the confined and strongly hydrogen bonded interfacial Im groups with electrode polarization at −0.8 V. Reflection adsorption IR (RAIR) spectra of SAMs with and without Im revealed that the bulky ring prevented the formation of a strongly hydrogen bonded amide group network.

## 1. Introduction

Histidine plays a crucial role in the architecture and activity of enzymes, as it is often found to ligate transition metal ions at the active sites of metalloproteins [1,2]. Such a versatility comes from its side chain imidazole (Im) ring that can take part in many interactions, for example (i) cation-π_Im_, (ii) π-π stacking, (iii) hydrogen_Im_-π, (iv) coordination with metal cations through nitrogen lone electron pair, and (v) hydrogen bonding (H-bonding) [2]. This versatility makes Im particularly suitable for surface applications through the SAM chemistry [3,4,5,6,7,8]. The Im-derivatives functionalized SAMs have been used in biosensing applications, modulations of enzyme activity, purification of biologically relevant molecules, corrosion prevention, and others [5,6,7,8].

The pK_a_ for imidazole is around 5.9. Thus, two forms of histidine side-chain functional group, Tautomer-I (T-I; N1–H, N3) and Tautomer-II (T-II; N1, N3–H), are possible under the physiological conditions. In neutral pH and room temperature, T-I is the more energetically favorable [9]. However, owing to the intermolecular Im interactions and the surface charge effect, the tautomeric equilibrium of surface-adsorbed Im derivatives might differ from that of the solution phase. Indeed, a clear transition from T-I to T-II was observed for Im functionalized lipoic acid compound as it adsorbed on the silver electrode [10]. Vibrational spectroscopy, especially Raman, is particularly useful in Im studies because of the diagnostic technique’s ability to differentiate between two tautomeric forms, probe ring protonation state and interactions with transition metal cations [9,10,11,12,13,14,15,16,17,18]. For example, the analysis of the three well-established Raman band pairs at 1568/1585, 1282/1260, and 983/1004 cm^−1^ allows to discriminate between T-I and T-II [19]. The coordination with metal ion effectively decreases the Im ring C4=C5 bond length, so that the related ν(C4=C5) increases by 5–20 cm^−1^ from the initial positions at 1568–1573 and 1583–1588 cm^−1^ for T-I and T-II, respectively [16]. The deuterium exchanged imidazolium cation (${\mathrm{ImD}}_{2}^{+}$) exhibits an intensive spectral mode near 1405 cm^−1^, which is particularly valuable in determining the imidazole deuteration state using regular Raman spectroscopy [20,21]. A special case to be mentioned is the investigation of Im ring deuteration state in proteins based on 1405 cm^–1^ spectral mode in UV-resonance Raman [22,23].

Surface-enhanced Raman spectroscopy (SERS) provides detailed molecular-level information on the bonding, orientation, and structure of surface-adsorbed molecules in situ [24,25,26,27,28,29]. However, SERS is restricted by the necessity to use corrugated noble metal surfaces. Recently developed shell-isolated nanoparticle-enhanced Raman spectroscopy (SHINERS) overcomes such a limitation [30,31]. The method is based on the Raman signal amplification by the plasmonic core nanoparticles (usually Au or Ag) that are covered with thin (2–3 nm) isolating shells from SiO_2_, TiO_2_, or other dielectric material. The inert shell protects the probed molecules from interactions with the metal core, increases nanoparticle stability, and acts as a barrier between the probe and the core, preventing from charge transfer and disturbance of the double layer. The SHINERS method has already shown great potential for the in-situ analysis of the molecule adsorption, interfacial structure, and interactions of molecules on smooth and well-defined surfaces and at electrochemical interfaces [32,33,34,35,36,37,38,39,40,41,42,43].

This research aims to attain molecular level insights into the structure and hydrogen bonding of the imidazole ring at the electrified interface. We report the synthesis and vibrational spectroscopy characterization of the imidazole ring terminated alkanethiol molecule with intrachain amide group, N-(2-(1H-imidazol-4-yl)ethyl)-6-mercaptohexanamide (IMHA). The use of in-situ electrochemical SHINERS and ex-situ reflection-absorption infrared spectroscopy (RAIRS) techniques allowed for probing IMHA SAM that was adsorbed on the highly defined, atomically smooth Au surfaces. The effect of electrode potential on monolayer structure, tautomerism, and hydrogen bonding interaction strength of imidazole ring at electrochemical interface was spectroscopically assessed.

## 2. Materials and Methods

### 2.1. Synthesis of N-(2-(1H-Imidazol-4-yl)ethyl)-6-mercaptohexanamide (IMHA) and 6-Mercapto-N-methylhexanamide (Fragment Molecule)

**Figure 1:**
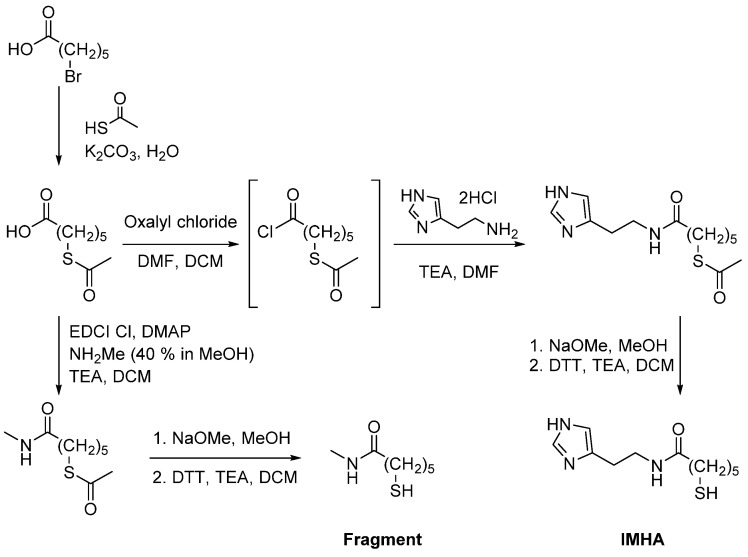
Synthesis scheme of IMHA and fragment molecule.

Materials were purchased from Apollo Scientific (histamine dihydrochloride), Alfa Aesar (6-bromohexanoic acid, oxalyl chloride, NaOMe (30 wt% in methanol)), TCI (1-(3-dimethylaminopropyl)-3-ethylcarbodiimide hydrochloride (EDCI Cl), methylamine (40% in methanol), and Sigma Aldrich (thioacetic acid, K_2_CO_3_, 1,4-dithio-DL-threitol (DTT), CH_2_Cl_2_ (DCM), methanol, triethylamine, dimethylformamide (DMF), 4-dimethylaminopyridine (DMAP)). DMSO-d6 (99.5 atom% D) and CDCl_3_ (99.8 atom% D) for nuclear magnetic resonance spectroscopy were obtained from Apollo Scientific. IMHA was synthesized from histamine dihydrochloride and 6-bromohexanoic acid, as shown in Figure 1. The details of synthesis and related analytical data of the IMHA, imidazole-truncated IMHA (6-mercapto-*N*-methylhexanamide) (fragment molecule), and their intermediates are presented in the Supplementary data file (General methods section, Scheme S1, and Figure S1).

### 2.2. Synthesis of Silicon Dioxide Covered Spherical Silver Nanoparticles (Ag@SiO_2_)

**Figure 2:**
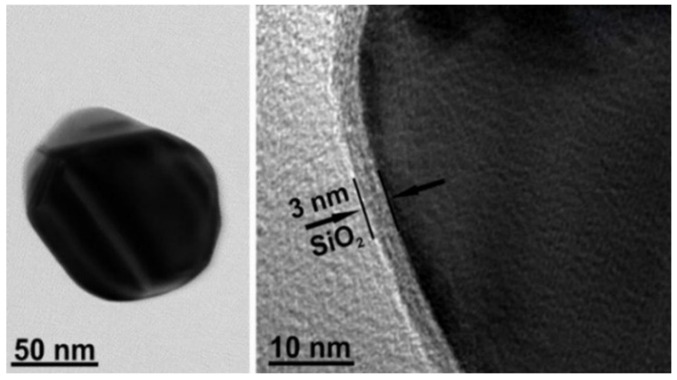
HR-TEM images of Ag@SiO_2_ nanoparticles. Ag core and SiO_2_ sizes were 85 ± 5 nm and 3 nm.

The reagents and solvents were used without further purifications: silver nitrate (AgNO_3_, 99%), trisodium citrate dihydrate (99%), (3-aminopropyl)triethoxysilane (99%), and sodium silicate solution (NaOH 10%, SiO_2_ 27%) (Merck). All solutions were prepared with ultra-pure water (resistivity of 18.2 MΩ·cm) from Direct-Q 3UV (Merck, Darmstadt, Germany). Nanoparticles (NPs) were synthesized under microwave irradiation according to the previously published method [44]. Briefly, the bare silver-core NPs were prepared by the AgNO_3_ reduction with sodium citrate and capped with silica shell. Then, core-shell NPs were purified by a repeated centrifugation, supernatant removal, and resuspension. Figure 2 shows the Ag@SiO_2_ NPs images obtained by high-resolution transmission electron microscopy (HR-TEM). HR-TEM images were collected by an FEI Tecnai G2 F20 X-TWIN TEM (FEI, Netherlands) microscope with an accelerating voltage of 200 kV. The microscope was equipped with an EDX (EDAX) spectrometer and Gatan Orius CCD camera. The measurements were carried out in a bright-field regime.

### 2.3. Preparation and Characterization of SAM

A 120-nm Au film was deposited on clean glass slides by using a Quorum 150T magnetron-sputtering machine and a 99.99% Au target. After that, the slides were incubated in 10^−3^ M IMHA ethanol solution for twenty-four hours unless indicated otherwise; then rinsed with ethanol and dried under N_2_. For SHINERS experiments, the slides were mounted into an electrochemical cell and filled with Milli-Q water. After that, 3 μL of Ag@SiO_2_ were carefully injected directly on the surface of Au and left to rest for 10 min for nanoparticles to adsorb. Then, the cell was thorough rinsed with 0.01 M phosphate buffer (pH 7) containing 0.1 M Na_2_SO_4_.

Reflection absorption IR spectroscopy (RAIRS) spectra were obtained by using a Vertex 80v FTIR spectrometer (Bruker, Germany) equipped with the LN-MCT narrow band detector and the horizontal reflection accessory. The spectral resolution was set at 4 cm^−1^. Spectra were acquired by 400 scans at a grazing angle of 80° by using p-polarized light. The sample chamber and the spectrometer were evacuated during the measurements to approximately 2 hPa pressure. The spectrum of deuterium substituted octadecanethiol (ODT-d38) SAM adsorbed on Au was used as a reference. FTIR transmission spectrum was recorded from KBr pellet-dispersed IMHA using an Alpha spectrometer (Bruker, Germany) equipped with an RT-DTGS detector. The resolution was set to 4 cm^−1^; 50 interferogram scans were co-added.

SHINERS spectra were recorded using high throughput (instrument NA = 0.22) Tornado HyperFlux spectrometer (Tornado Spectral Systems, Mississauga, ON, Canada) equipped with fiber-optic cable for excitation and collection of the Raman spectra. The 785 nm beam of the diode laser was used as the excitation source. The laser power at the sample was set to 38 mW, and the beam was focused on a 100 μm diameter spot on the sample. Spectra were recorded for 300 s by co-adding thirty 10-s scans. For temperature-controlled Raman measurements, the LinKam temperature control system PE95/T95 with the accuracy of 0.05 °C was used. IMHA powder was measured with 80 mW laser power and 100 s integration time, at 22, 130, and 135 °C. Spectroelectrochemical measurements were carried out in a three-electrode cell, where slides with Au film were used as a working electrode, platinum wire as a counter electrode, and KCl saturated Ag/AgCl as a reference electrode.

Raman wavenumbers were calibrated according to the polystyrene spectrum. Band frequencies were obtained by fitting experimental contours with mixed Gaussian-Lorentzian form components by using the GRAMS/AI 8.0 (Thermo Scientific, Waltham, MA, USA) software. 

Theoretical modeling study of IMHA molecule was performed using Gaussian package version G09 D.01 [45]. Geometry optimization and frequency calculation were completed with the DFT method using the hybrid B3LYP functional and 6-311++G(2d,p) basis set. The polarizable continuum model (IEFPCM) was used to emulate the water environment. The frequency calculation ended with no imaginary wavenumbers, indicating the geometry at minimum energy. Frequency and intensity scaling were applied according to the previously described procedure [46].

## 3. Results and Discussion

### 3.1. Assignments of Raman Bands

**Figure 3:**
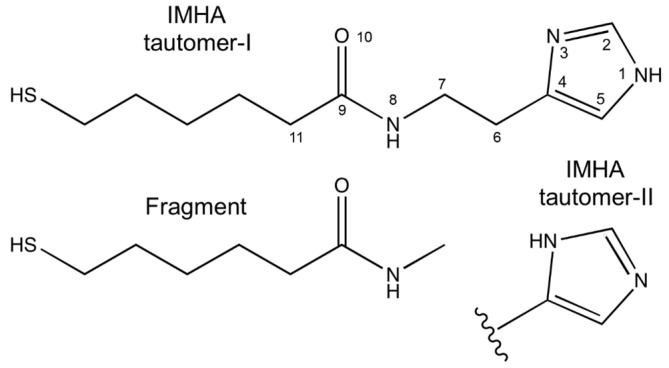
Molecular structure of Tautomer-I and Tautomer-II form of IMHA and the structure of fragment molecule.

The studied IMHA molecule comprises four functional units: (i) the surface-active thiol group (SH), (ii) the hydrocarbon chain (−(CH_2_)_5_−), (iii) the amide group (−CO−NH−), and (iv) the terminal Im ring (Figure 3). To facilitate Raman band assignments, we have synthesized a compound similar to IMHA thiol without the terminal Im ring (fragment compound) (Figure 3). Detailed assignments of the Raman bands are provided in the Supplementary Data file (Figure S2 and related discussion) and Table 1.

**Figure 4:**
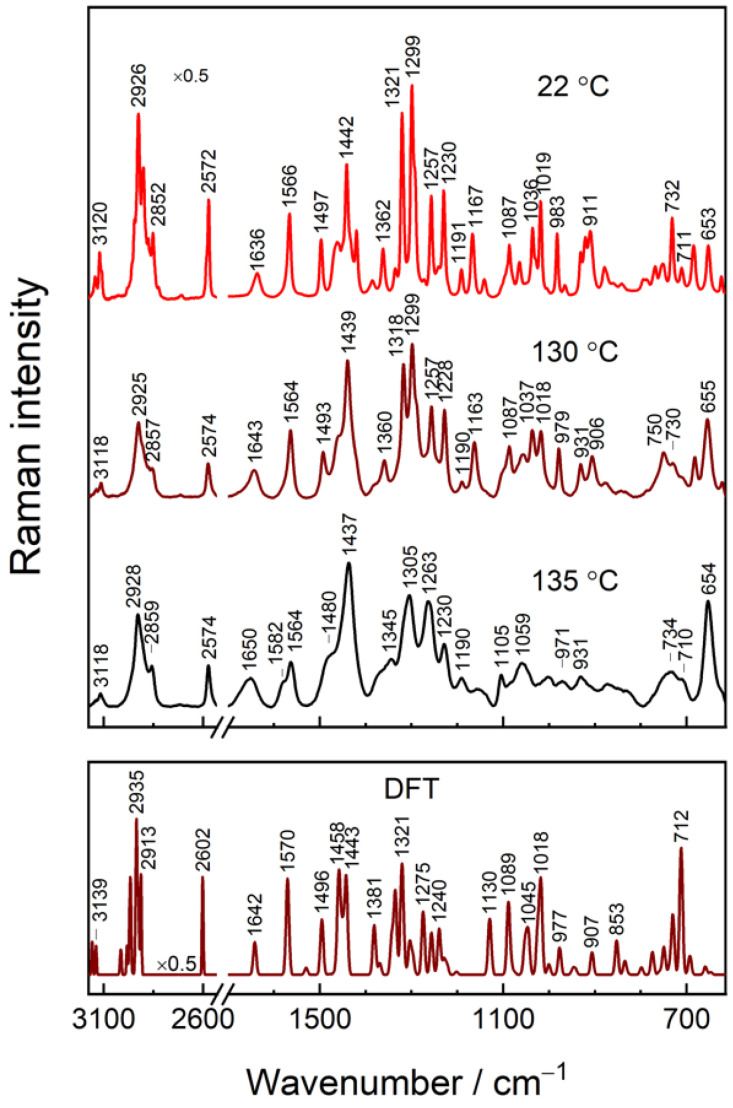
Temperature-dependent Raman spectra of solid IMHA at 22, 130, and 135 °C (upper panel) and DFT spectrum of IMHA (bottom panel). The intensity of the 2530–3175 cm^−1^ region is scaled by 0.5.

To evaluate Raman modes related to intra- and intermolecular interactions and those sensitive to the ordering of alkyl chain, we performed a temperature-dependent Raman study of powder IMHA compound (Figure 4). Changes in temperature provoke structural or phase transitions and a variety of conformational alterations in a molecular system. In general, an increase in temperature induces disordering in the molecular arrangement. Such disordering may be reflected in changes of spectral mode bandwidths (related to vibrational energy distribution), peak position (energy of particular vibration), and relative intensities (distribution of two or more different forms of a molecule). Therefore, temperature-Raman provides additional means for scrutinizing a given compound. The medium intensity modes near 1566, 1321, and 983 cm^−1^ in the 22 °C spectrum are related to vibrations of the Tautomer-I (N1–H, N3) form of Im ring and are assigned to ν(C4=C5) stretching, ν(Im) breathing + δ(C5H), and β(CH) deformation, respectively [19]. At the elevated temperatures (130–135 °C), the shoulders appear at 1582 and 1345 cm^−1^ specific to the Tautomer-II (N1, N3–H) form of Im. The relative percentage of T-II determined by the integral intensity ratio A_1582_/(A_1582_ + A_1564_) is 16% for the compound at 130 °C and 32% for the one at 135 °C. It should be noted that the frequency of an intense band at 1321 cm^−1^ downshifts to 1305 cm^−1^ at 135 °C. In addition, the band notably broadens. Such spectral changes reflect a decrease in H-bonding interaction strength primarily at N3 site, because of a considerable contribution from ν(N3−C2) vibration to this mode for Tautomer-I [19].

The 1497 cm^−1^ band assigned to ν(N1–C2) + β(C2H) motion is not sensitive to Im tautomerism [19]. The band, however, shifts to higher wavenumbers with the coordination of metal cation at the nitrogen lone electron pair. Moreover, electrode polarization also induces shifting for imidazole-copper pair (9 ± 2 cm^−1^V^−1^) [18]. We found a 17 cm^−1^ downshift of the spectral mode to 1480 cm^−1^ and a clear increase in full width at half-maximum (FWHM) with the temperature raised to 135 °C. These spectral changes are associated with the increased motional freedom of the Im ring. Therefore, the spectral changes near 1496–1480 cm^−1^ may serve as a useful spectral marker for Im ring packing.

Nested within the alkane chain, the amide group acts as an intermolecular stabilizing agent, which forms the extended H-bond network between neighboring molecular chains. It has been already shown that such an interaction radically increases the desorption temperature and chemical stability of the monolayer [50]. From the temperature-Raman spectra of the powder compound, only the medium-low intensity Amide-I (Am-I) band at 1636 cm^−1^ can be recognized. This spectral mode is related to C=O stretching (83%) coupled with out-of-phase ν(C−N) and δ(C–C−N) and can serve as a diagnostic tool in identifying the secondary structure of peptides [47]. The 14 cm^−1^ frequency upshift during the solid-to-liquid transition with the temperature elevation, clearly shows the weakening of the H-bonding at the amide’s C=O group.

### 3.2. RAIRS Analysis of the Monolayer Formation

**Figure 5:**
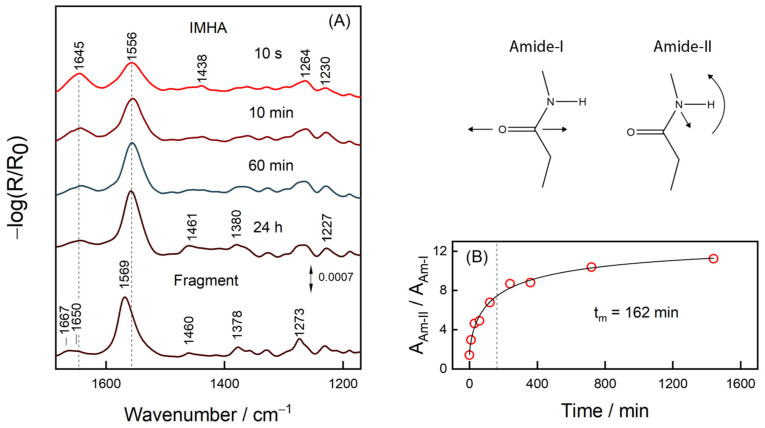
(**A**) The immersion time dependent RAIRS spectra of IMHA and the spectrum of the fragment molecule after 24 h-incubation. (**B**) Dependence of integral intensity ratio Am-II/Am-I on the immersion time fitted with the sigmoidal curve (R^2^ = 0.9925). The transition midpoint at 162 min marked by a dashed line. Cartoon depicts the atom motions in Amide-I and Amide-II vibrations.

Figure 5 shows RAIRS spectra of the IMHA monolayer adsorbed on a smooth gold surface. The spectral features at 1645, 1556, and 1264 cm^−1^ are assigned to amide bands, Am-I, Am-II, and Am-III, respectively, whereas the ones near 1460 and 1380 cm^−1^ to methylene scissoring and wagging deformations, δ(CH_2_). The RAIRS-surface selection rule allows interrogating the orientation of molecular groups because the intensity of particular spectral mode directly depends on the projection of the mode’s transition dipole moment (TDM) on the surface normal [51]. While Am-I and Am-II have TDMs oriented perpendicular to each other in the amide bond plane, they also have perpendicular and parallel TDM orientations with respect to carbohydrate chain [51]. Thus, for the neatly packed IMHA monolayer, the Am-II is expected to dominate the spectrum. Indeed, at progressively longer incubation time, the Am-II intensity at 1556 cm^−1^ increases and the intensity of Am-I at 1645 cm^−1^ decreases. The integral intensity ratio Am-II/Am-I provides a qualitative measure of the molecular reorientation (Figure 5B). The experimental data of Am-II/Am-I were fitted with a sigmoidal function:

(1) $$A=A_{0}+\frac{a}{1+{\left(\frac{t}{t_{m}}\right)}^{b}}$$

where *t_m_* is the transition inflection point found at 162 min. At 10 s incubation time, the Am-II/Am-I ratio was 1.4, which is slightly above 0.9 calculated for IMHA molecules chaotically dispersed in KBr pellet (Figure S3). The ratio doubled in 10 min and after 24 h it reached 11.2, so most molecules had a planar amide group oriented perpendicularly to the surface. Such an orientation is strengthened by the H-bonding interaction involving the amide group (C=O···H) and dipole–dipole interaction between the C=O groups [52]. The link between Amide-I and -II wavenumbers and the H-bonding strength at C=O and N–H groups has been clearly established [53,54,55]. We find marginal Am-I frequency decrease with the development of SAM (by 3 cm^−1^ during the 120 min incubation) revealing minor increase of the H-bonding strength at C=O. The frequency of the Am-II band remained constant.

In the RAIRS spectrum of 24 h-incubated fragment SAM, the Amide-II spectral band is blue-shifted by 12 cm^−1^ compared to the IMHA SAM (after 24 h incubation), showing the stronger H-bonding at the N–H group for fragment molecule. Interestingly, surface-adsorption of molecules from solution phase induced spectral shift of Am-II mode by −20 for IMHA and 13 cm^−1^ for the fragment molecule (Table 2). Such frequency shifting is related to different molecular packing efficiency in bulk and SAM. The Im ring in IMHA molecule introduces sterical hindrances for the neighboring amides to engage in the formation of an optimal H-bonding network.

### 3.3. General Features of IMHA Monolayer SHINERS Spectrum

**Figure 6:**
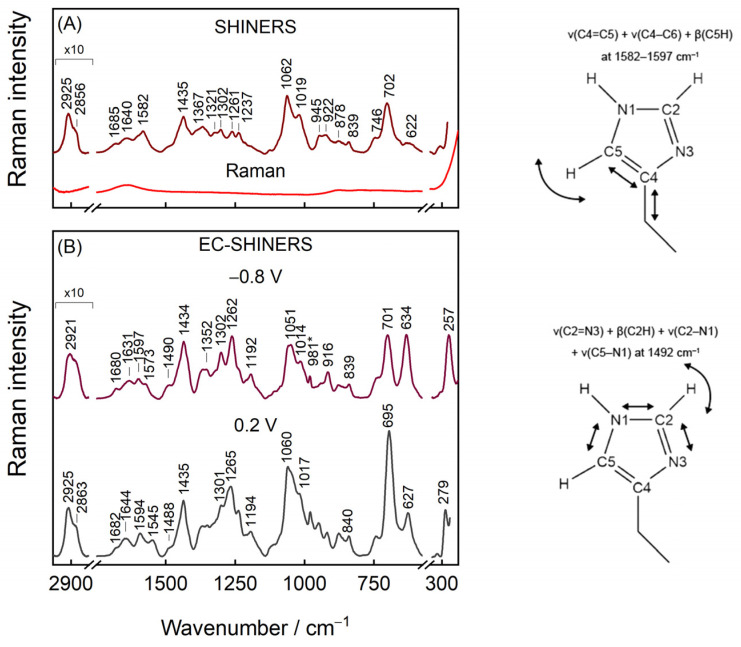
(**A**) SHINERS and Raman spectra of IMHA SAM on a smooth Au electrode in H_2_O. (**B**) EC-SHINERS spectra recorded at −0.8 and 0.2 V potentials in phosphate buffer solution (PBS; pH 7.0, with 0.1 M Na_2_SO_4_). Asterisk (*) at 981 cm^−1^ marks SO_4_^2−^ vibrational mode from the solution. Cartoon depicts the selected imidazole ring motions.

Figure 6A presents evidence of Ag@SiO_2_ functionality. The Raman spectrum of nanoparticles has virtually no vibrational modes until the nanoparticles are placed on top of smooth surface-adsorbed IMHA monolayer. The intense mode at 702 cm^−1^ corresponds to ν(C–S)_T_ stretching vibration of the molecules, which adopt a nearly vertical orientation with the surface at the C–S bond, whereas the corresponding gauche mode ν(C–S)_G_ appears as a weak feature at 622 cm^−1^. The predominant trans configuration agrees with the vertical orientation of molecules at the amide group in mature SAM as revealed by RAIRS. Weak Am-I bands at 1685 and 1640 cm^−1^ were immediately ascribed to surface molecules that are involved in strong and weak H-bonding at amide groups, respectively. The rather complex 1500–1700 cm^−1^ region contains no Am-II mode because this mode is typically either very weak or not detectable in Raman spectra [56]. Two strong bands at 1062 and 1019 cm^−1^ were found to be not sensitive to H/D exchange and were assigned to stretching vibrations of hydrocarbon chain, ν(C–C)_T_ and ν(C–C), respectively (Table 1). The higher frequency component was assigned to the in-phase vibration of –(CH_2_)_5_− chain in extended all-trans conformation based on DFT calculations (1089 cm^−1^ band) and previously reported temperature-dependent SERS studies of SAMs [57]. The 1019-cm^−1^ band was assigned to the C–C stretching vibration of hydrocarbon chain connecting amide and Im groups based on DFT suggestion (1018 cm^−1^ band).

The tautomeric equilibrium of Au-adsorbed IMHA could certainly differ from that of the solution phase. Indeed, ν(C4=C5) + ν(C4–C6) + β(C5H) mode at 1582 cm^−1^ fall close to the 1588−1583 cm^−1^ range typical for T-II [16]. While in the case of T-I conformer, this mode is expected to be observed at considerably lower wavenumbers (1573–1568 cm^−1^). The downshift of this mode to 1575 cm^−1^ in D_2_O solution immediately confirms H/D exchange at nitrogen sites of Im ring in the monolayer. Another couple of bands, at 1261 for H_2_O and 1257 cm^−1^ for D_2_O solutions, confirm the T-II surface form of IMHA [16].

### 3.4. Potential-Controlled SHINERS Measurements of IMHA Monolayer

Negative electrode polarization at −0.8 V potential was applied for 30 s in the beginning of each potential-controlled measurement to desorb impurities of low surface affinity, which may originate from nanoparticles. Preliminary examination showed that pretreatment of the monolayer at negative potentials increases the reproducibility of results. Figure 6B shows SHINERS spectra from the smooth Au electrode adsorbed IMHA at −0.8 and 0.2 V electrode polarizations.

Plenty of SERS research on metal–sulfur bond has been carried out for the roughened metal adsorbed monolayers [57,58]. However, only with SHINERS approach the well-defined and smooth substrate adsorbed molecules become more accessible to Raman spectroscopy [37,59]. The Au–S stretching mode found at 257 cm^−1^ upshifts to 279 cm^−1^ (δ = 22 cm^−1^) when the potential is tuned from −0.8 to 0.2 V attesting the increase in Au–S bond strength. A similar value was found in a previous study on flat Au-adsorbed N-(6-mercapto)hexylpyridinium [37], whose high tuning rate was ascribed to force constant changes in Au–S bond strength because of electrode polarization and to some extent to the Stark effect [37,60]. As for C–S bond, the populations of gauche and trans molecular conformers were matching at −0.8 V, but with positive electrode polarization trans became strongly favored (Figure S4). Both conformational bands redshift with the tuning rate of −5.3 ± 0.2 and −6.9 ± 0.2 cm^−1^V^−1^, respectively, indicating potential-induced decrease in C–S bond strength, while at the same time Au–S bond became increasingly stronger at more positive potentials [61]. Within the tested potential window, the trans/gauche ratio (I_701_/I_633_) increases from 1 to 3 with transition midpoint potential of −0.34 ± 0.02 V. Notably, all spectral changes are reversible as the electrode potential is set to −0.8 V. Two strong bands at 1051 and 1014 cm^−1^ were assigned to stretching vibrations of hydrocarbon chain, ν(C–C)_T_ and ν(C–C), respectively (Table 1). It should be noted that the relative intensity of ν(C−C)_T_ band increases at more positive electrode potentials together with growing ν(C−S)_T_ mode. In the high frequency region of Figure 6B, symmetric and asymmetric stretching vibrations of methylene groups ν(CH_2_) near 2858 and 2921 cm^−1^ were found (E = −0.8 V). These modes will be discussed in more detail later in the manuscript.

**Figure 7:**
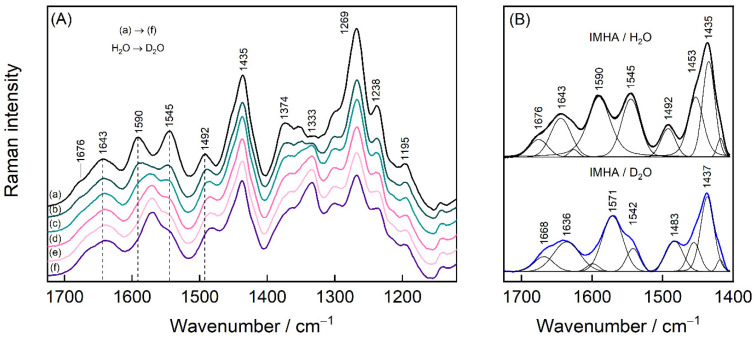
(**A**) SHINERS spectra in 1120–1725 cm^−1^ region of IMHA monolayer at open circuit potential in PBS solution (a), which was gradually exchanged to D_2_O (b)–(f). In each step, 20 vol% of the solution was removed from the cell and then the same amount of D_2_O was added. (**B**) The spectra of the first and the last step of the H/D exchange fitted with Gaussian-Lorentzian shape components.

To interpret the rather complex midrange region that is occupied by the imidazole ring, amide group vibrations, and the deformations of the methylene groups, a separate H/D exchange experiment was performed for the IMHA monolayer. The H/D exchange process was accomplished at the open circuit potential (0.17 V) by exchanging phosphate buffer solution (PBS) (with 0.1 M Na_2_SO_4_, pH 6.9) to pure D_2_O (Figure 7). Amide-I spectral mode is found to be composed of low- and high-energy components at 1643 and 1676 cm^−1^, both of which downshift by 7–8 cm^−1^ upon the exchange. We find that vibrational modes in the 1300–1610 cm^−1^ were sensitive to the solvent exchange. For example, the Tautomer-II related C=C stretching mode of the Im ring ν(C4=C5) clearly downshifts from 1590 to 1571 cm^−1^ [16]. The well-defined feature at 1492 cm^–1^ assigned to ν(C2–N3) coupled with β(C2H) downshifts to 1483 cm^−1^ in accordance with literature data [18]. The intense band at 1333 cm^−1^ develops in D_2_O solutions. This band was identified as a Tautomer-II marker band for N3D protonated histidine [62]. Interestingly, the mode at 1545 cm^−1^ becomes significantly pronounced at 0.2 V potential (Figure 7A) and almost disappears due to the H/D exchange. Its assignment remains not fully clear because the contribution from the Am-II could be almost certainly ruled out since the deuteration of amide’s N atom would introduce Am-II’ with additional spectral intensity near 1450 cm^−1^ [63,64]. The only possible proximal spectral modes are expected near 1535 cm^−1^ for deprotonated 4-methylimidazole and doubly protonated histidine, both of which in neutral pH are not likely [13,62]. Presented SHINERS data revealed that the imidazole ring in the SAM at relatively positive electrode potentials is in the N3H protonation state (Tautomer-II).

**Figure 8:**
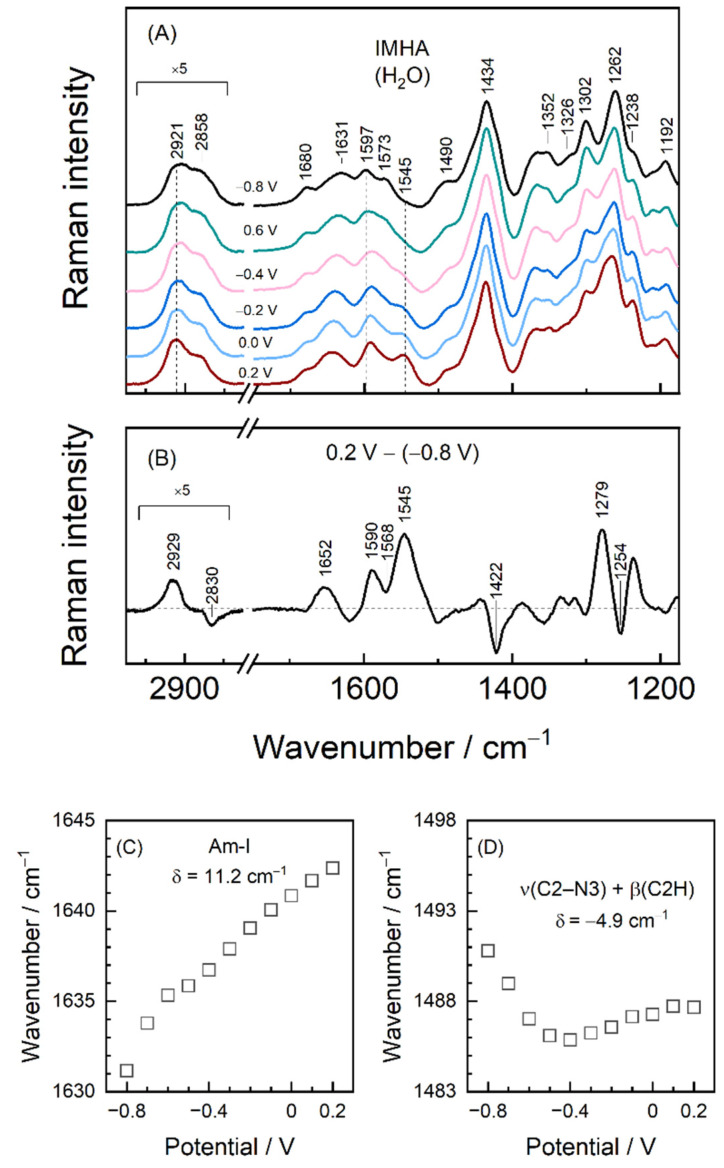
(**A**) EC-SHINERS spectra in 1120–1750 cm^−1^ and 2750–3050 cm^−1^ regions at indicated potentials. Spectra correspond to the ones in Figure 6. (**B**) EC-SHINERS difference spectrum constructed by subtracting 0.2 V spectrum from the one registered at −0.8 V. (**C**) Potential dependence of the Amide-I and (**D**) ν(C2–N3) + β(C2H) mode wavenumbers.

Figure 8 shows the detailed SHINERS analysis of biased potential induced structural changes in IMHA SAM. An 11.2 cm^−1^ upshift in the frequency of dominant Amide-I component is detected with more positive electrode potentials (Figure 8C). To complement that, we analyzed the relative intensities of Am-I modes of strongly (ca. 1631 cm^−1^) and loosely H-bonded (ca. 1680 cm^−1^) molecules. The intensity ratio of these expressed as I_1631_/I_1680_ monotonically decreased from 3.4 to 2.3 with the negative to positive potential excursion. Both frequency shift and relative intensity changes show that a positive bias potential disengages molecules from the more strongly H-bounded network at the C=O moiety of amide groups.

As Figure 6 and Figure 8 show, the positions of imidazole-tautomerism marker bands near 1262, and 1597 cm^−1^ remain consistent with N3–H protonation (Tautomer-II) within the tested potential window [19]. However, at −0.8 V potential, a shoulder at 1573 cm^−1^ associated with Tautomer-I protonation form of Im becomes visible. Positive polarization diminishes mode intensity, while its T-II counterpart at 1597 cm^−1^ becomes stronger. Thus, SHINERS data show the T-II being the preferred tautomeric form at each tested potential, with a minor portion of molecules adopting T-I at the most negative polarizations. Potential induces a slight shift of the 1597 cm^−1^ to lower wavenumbers by 3.6 cm^−1^ due to lengthening of the C4=C5 bond. Interestingly, the Im ring mode at 1490 cm^−1^ assigned to C2–N3 stretching coupled with in-plane deformation β(C2H), ν(C2–N1), and ν(C5–N1) shows the nonmonotonous dependency on potential [10,18]. This mode shifts by −4.9 cm^−1^ (tuning rate 13.3 cm^−1^V^−1^) in the range from −0.8 to −0.4 V and then upshifts by 1.8 cm^−1^ (Figure 8D). Besides, the spectral mode notably increases in FWHM by ca. 10 cm^−1^ of the −0.4 V spectrum compared to the −0.8 V one. From temperature-Raman measurements, decreasing mode frequency and increasing FWHM were linked with liberated imidazole ring motion. Thus, spectral data show that Im ring confinement in the monolayer at more positive electrode potentials is relaxed. The strong band at 1262 cm^−1^ (−0.8 V) due to Im ring breathing vibration coupled with C2–H in-plane deformation is very sensitive to potential perturbation; frequency of this band upshifts to 1266 cm^−1^ at electrode polarization 0.2 V. Consequently, the potential-difference spectrum clearly shows derivative-like feature at 1279/1254 cm^−1^ (Figure 8B). Temperature-dependent Raman study of bulk IMHA compound revealed an upshift in the position of this mode at elevated temperature (135 °C) (Figure 4). This might be related to decreased H-bonding interaction strength at the Im ring site. Such spectral observations are consistent with decreased H-bonding interaction strength at both amide (Am-I band) and Im ring sites at more positive electrode polarization.

### 3.5. Raman Markers for H-Bonding Interaction

**Figure 9:**
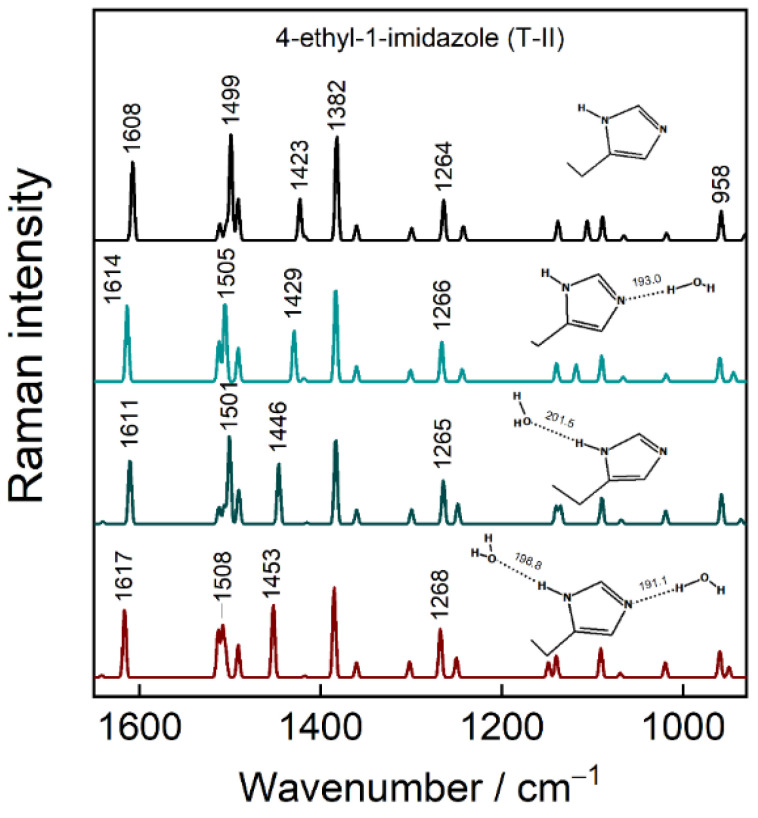
DFT spectra and optimized structures of Tautomer-II form of model compound 4-ethyl-1-imidazole with H-bonding coordination number from 0 to 2. The lengths of H-bonds are indicated in pm.

In order to better understand the way H-bonding interaction affects imidazole ring structure and its vibrational frequencies, DFT modeling was carried out for 5-ethyl-1H-imidazole (Im-CH_2_-CH_3_) molecule isolated in vacuum and coordinated with one and two explicit water molecules (Figure 9). Calculations of T-II form molecule predict that 1608-cm^–1^ mode is mainly C4=C5 stretching motion; 1499 cm^−1^ mode is assigned to ν(C2–N1) + ν(C2–N3) + β(C2H) and associated with 1492 cm^−1^ mode in SHINERS spectra. The 1423 cm^–1^ mode emerges due to β(N3H) + ν(N3–C4) + ν(N3–C2) vibrations; 1382 cm^−1^ mode is tautomerism sensitive Im breathing mode. With increasing H-bonding coordination number, the 1499-cm^−1^ mode shifts to higher frequencies by 2–6 cm^−1^ (coordination number 1) and by 9 cm^−1^ (coordination number 2). The same holds for a compound in T-I form, for which an 8 cm^–1^ shift was found when in contact with two H_2_Os. The predicted wavenumber shift confirms the principal mode’s sensitivity to H-bonding interaction at the Im group and agrees with the temperature-Raman data presented in Figure 4 where the higher wavenumbers were associated with stronger hydrogen bonding interactions for the sample at room temperature. We already ascribed the decrease in position of 1492 cm^−1^ mode with liberated Im ring based on temperature-Raman. However, DFT proves that such a liberation is hydrogen bonding strength-related. Thus, the sharp wavenumber drop from −0.8 to −0.4 V potentials in Figure 8D clearly indicates reduction of H-bonding at Im, which is followed by a slight strengthening in −0.4/0.2 V range.

Interestingly, we found stronger H-bonding interactions for Im coordinated with two H_2_O molecules rather than one H_2_O. In this case, the length of N1···H_2_O and N3–H···H_2_O bonds decreased by 1.9 and 2.7 pm with an introduction of the second H_2_O molecule (insets in Figure 9). A much higher, up to 30 cm^−1^, frequency shift was found for 1423-cm^−1^ mode. However, this mode was difficult to identify in experimental SHINERS spectra of IMHA SAM because of the overlapping with methylene deformation vibrations.

## 4. Conclusions

Hydrogen bonding is fundamental in protein architecture, interactions, and molecular recognition. Imidazole group of histidine amino acid is a major player in active centers of enzymes and protein secondary structure because of its ability to accept and donate H-bonding. To study H-bonding interactions of imidazole ring at electrified interfaces, first, we synthesized alkanethiol molecule with interchain amide group and terminal imidazole functional group (IMHA), then studied IMHA SAMs adsorbed on atomically smooth Au surfaces by means of SHINERS and RAIRS techniques.

(i) From RAIRS data molecules adsorb on a surface chaotically but with time (transition midpoint was 162 min), their intrachain amide groups become nearly perpendicular to the surface, which points to the more neatly organized SAM. However, the formation of an optimal H-bond network among amide groups was precluded by the steric hindrances introduced by bulky IMHA’s imidazole group, as revealed by a study of the imidazole-truncated fragment molecule. For further endeavors, mixed IMHA/fragment molecule SAMs are suggested.

(ii) Adsorption on a surface induced a tautomeric transition in IMHA molecules from Tautomer-I (N1–H, N3) to Tautomer-II (N1, N3–H). The Tautomer-II was strongly favored at electrode polarization close to open circuit potential. However, an increased portion of Tautomer-I molecules was observed at −0.8 V.

(iii) Temperature-Raman and DFT modeling identified the 1492 cm^−1^ mode as sensitive to imidazole ring confinement and H-bonding strength. The analysis of this mode revealed the potential-dependent behavior of the interfacial Im ring, such that the Im is the most strongly confined and H-bonded at −0.8 V electrode polarization and the weakest confined and H-bonded at −0.4 V.

The presented data on construction, structure, and potential dependence of imidazole ring group-functionalized SAMs will possibly be valuable for the development of biosensors, molecular electronics, environmental, and pollution studies.

## Figures and Tables

**Table 1:** Temperature-Raman, SHINERS, and DFT frequencies and assignments of IMHA.

Raman, cm^−1^			SHINERS, cm^−1^	DFT	Ref.	Assignment
22 °C	130 °C	135 °C	H_2_O, −0.8 V			
3142	3139	3144 sh		3158		ν(C5–H)
3120	3118	3118		3139		ν(C2–H)
2926	2925	2928	2921	2935		ν_as_(CH_2_)
2852	2857	2859	2856	2913		ν_s_(CH_2_)
2572	2574	2574	n.a.	2602		ν(S–H)
1636	1643	1650	1680 1631	1642		ν(C=O) Amide-I
		1582 T-II	1597 T-II	1587 T-II	[9,18,19]	ν(C4=C5) + ν(C4–C6) + β(C5H)
1566 T-I	1564 T-I	1564 T-I	1573 T-I	1570 T-I		
				1530		ν(C–N) + δ(NH) Amide-II
1497	1493	1480	1490 sh	1496	[10,18]	ν(C2–N3) + β(C2H) + ν(C2–N1) + ν(C5–N1)
1464	1461			1458		δ(CH_2_) scissoring
1442	1439	1437	1434	1443		δ(CH_2_) scissoring
1362	1360		1372	1381		w(CH_2_)
		1345 T-II	1352 sh T-II		[19]	δ(CH_2_) + ν(Im) breathing + δ(C5H)
1321 T-I	1318 T-I		1326 sh T-I	1335 T-I		
1299	1299	1305	1302	1303		t(CH_2_)
1257 T-II	1257 T-II	1263 T-II	1262 T-II		[9,19,47]	ν(Im) breathing + β(C2H)
1230	1228	1230	1238	1240	[18]	β(C5H) + β(C2H) + ν(C5–N1)
1191	1190	1190	1192	1202		t(C6H_2_) + δ(N8H)
1167	1163			1130	[13]	ν(C2–N1) + δ(N1H)
1087	1087	1105		1089		ν(C–C)_T_ + δ(CSH) + δ(CCS)
1036	1037	1059	1051	1045		ν(C–C)_T_
1019	1018		1014	1018		ν(C6–C7)
983	979	971		977	[9,19]	β(CH) Im for T-I
931	931	931		943		t(CH_2_) + r(CH_2_)
921			916	948	[13]	β(CH) Im
911	906			907		δ(N8C9C11)
841	842		839	835		γ(C2H)
753	750			750	[48]	γ(C5H) + r(CH_2_)
732	730	734		730	[48]	r(CH_2_) + ν(S–C)_T_
711	709	710	701	712		ν(S–C)_T_
685	682			694		γ(Im)
653	655	654	634	n.a.	[49]	ν(S–C)_G_ + δ(Im)

Abbreviations: n.a., not applicable; sh, shoulder; G, gauche; T, trans; r, rocking; w, wagging; t, twisting; δ, deformation; β, in-plane deformation; γ, out-of-plane deformation; ν, stretching; Im, imidazole; T-I, Tautomer-I; T-II, Tautomer-II.

**Table 2:** Wavenumbers and FWHM (bold) of Am-I and Am-II modes of IMHA and fragment molecule obtained from samples in powder form and SAMs.

	IMHA			Fragment		
	SAM, 24 h	Powder	*δ*	SAM, 24 h	Powder	*δ*
Am-II, cm^−1^	1557, **29**	1577, **37**	−20	1569, **28**	1556, **52**	13
Am-I, cm^−1^	1642, **42**	1638, **27**	6	1650, **28;** 1667, **16**	1647, **41**	3; 20

Abbreviation: δ, wavenumber shift, ν_SAM_–ν_powder_.

## Data Availability

All data supporting the findings of this study are available from the corresponding author upon reasonable request.

## Supplementary Materials

The following supporting information can be downloaded at: https://www.mdpi.com/article/10.3390/molecules27196531/s1, Scheme S1: Synthesis scheme of IMHA compound and fragment molecule; Figure S1: 1H NMR and 13C NMR spectra of N-(2-(1H-imidazol-4-yl)ethyl)-6-mercaptohexanamide (IMHA); Figure S2: (A) Raman spectra of powder IMHA, fragment molecule, and histidine. (B) Raman spectra of powder fragment molecule and the 0.33 M solutions in H_2_O and D_2_O. Figure S3: FTIR transmission spectra of IMHA and fragment molecule powders dispersed in KBr pellets. Am-I and Am-II spectral modes were approximated using Gaussian shape components. The full-widths at half maxima of Am-I and Am-II bands are given next to corresponding wavenumbers; Figure S4: (A) SHINERS spectra of IMHA adsorbed on smooth Au electrode at indicated potentials in the 600–750 cm^–1^ spectral region. Spectra were recorded in 0.1 M Na_2_SO_4_ aqueous solution containing 0.01 M phosphate buffer (pH 7). The excitation wavelength was 785 nm. (B) Dependence of relative integrated intensity I_701_/I_633_ ratio of IMHA bands on electrode potential fitted with the sigmoidal curve (Boltzmann model, R^2^= 0.99658). The transition midpoint potential value was determined at –0.34 ± 0.02 V, shown by the dashed line. The grey bar around the midpoint line shows the error in the fitting value. (C) Dependence of ν(C–S)_G_ and ν(C–S)_T_ modes wavenumbers of IMHA on electrode potential. Data in (B) and (C) sections were presented as an average of three independent measurements.

## Sample Availability

Samples of the compounds are available from the authors.
