# Supplementary material for: Electrochemical Shell-Isolated Nanoparticle-Enhanced Raman Spectroscopy of Imidazole Ring Functionalized Monolayer on Smooth Gold Electrode
Source: Molecules. 2022 Oct 3;27(19):6531. doi: 10.3390/molecules27196531 (PMC9573715; doi:10.3390/molecules27196531)
Supplement: Supplementary file 1 [file molecules-27-06531-s001.zip › molecules-1902736-supplementary.pdf]

Supplementary Data

Electrochemical Shell-Isolated Nanoparticle-Enhanced Raman Spectroscopy of Imidazole Ring Functionalized Monolayer on Smooth Gold Electrode

Agnė Zdaniauskienė [1], Martynas Talaikis [2,*], Tatjana Charkova [1], Rita Sadzevičienė [1], Linas Labanauskas [1], Gediminas Niaura [1,2,*]

[1] Center for Physical Sciences and Technology (FTMC), Department of Organic Chemistry, Sauletekio Ave. 3, LT-10257 Vilnius, Lithuania

[2] Vilnius University, Life Sciences Center, Institute of Biochemistry, Department of Bioelectrochemistry and Biospectroscopy, Sauletekio Ave. 7, LT-10257 Vilnius, Lithuania

*correspondence: martynas.talaikis@gmc.vu.lt; gediminas.niaura@ftmc.lt

Materials and Methods

Synthesis and characterization of N-(2-(1H-imidazol-4-yl)ethyl)-6-mercaptohexanamide (IMHA) and 6-mercapto-N-methylhexanamide (Fragment molecule)

General methods

Compounds NMR spectra were recorded on a Bruker Ascend 400 spectrometer in DMSO-d6 or $CDCl_3$. Chemical shifts are reported in ppm relative to solvent resonance signal as an internal standard. Melting points were recorded in open capillary using Mettler Toledo FP90 Central processor equipped with Mettler Toledo FP81HT MBC Cell and are not corrected. Chromatography was performed using Apollo Scientific Zeoprep 60 35-70 µm silica gel for flash chromatography and Merck TLC Silica gel 60 F_{254} plates for TLC. Starting materials were purchased from Apollo Scientific (histamine dihydrochloride), Alfa Aesar (6-bromohexanoic acid, oxalyl chloride, NaOMe (30% w/w in methanol)), TCI (1-(3-dimethylaminopropyl)-3-ethylcarbodiimide hydrochloride (EDCI Cl), methylamine (40% in methanol, ca 9.8 mol/L) and Sigma Aldrich (thioacetic acid, potassium carbonate, 1,4-dithio-DL-threitol (DTT), dichloromethane, methanol, triethylamine, dimethylformamide (DMF), 4-dimethylaminopyridine (DMAP)). DMSO-d6 (99.5 atom% D) and $CDCl_3$ (99.8 atom% D) for NMR spectroscopy were obtained from Apollo Scientific.

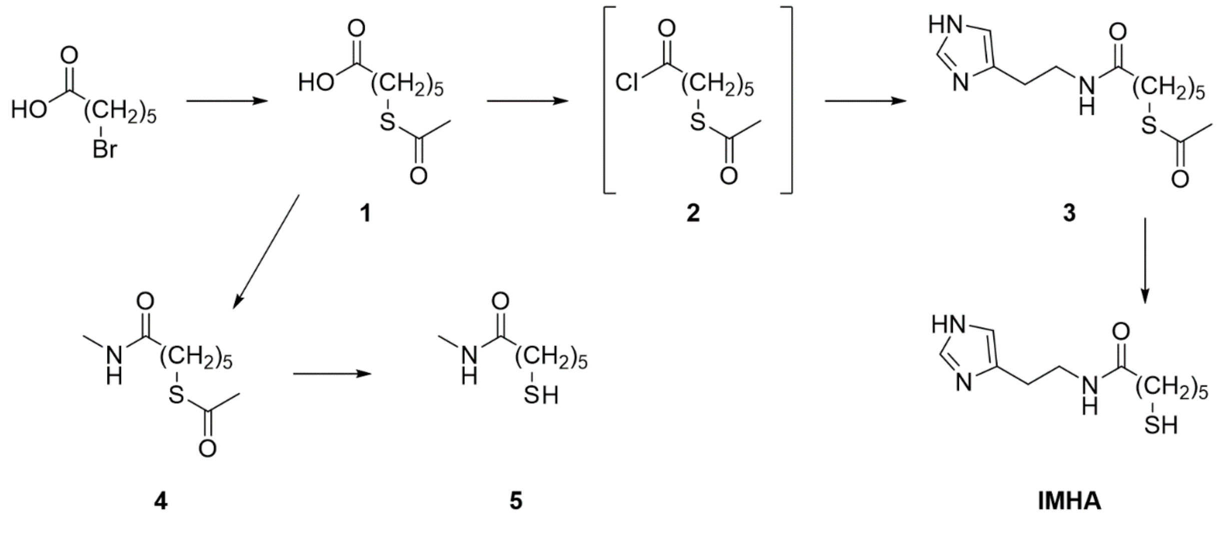

Scheme S1. Synthesis scheme of IMHA compound and fragment molecule **5.**

6-(acetylthio)hexanoic acid (1)

To a solution of K_2CO_3 (9.2 g, 66.6 mmol) in deionized water (30 mL), thioacetic acid (4.3 mL, 61.5 mmol) was added dropwise at 10 °C under argon atmosphere. After 30 min solution of 6-bromohexanoic acid (10 g, 51.3 mmol) and K_2CO_3 (7.09 g, 51.3 mmol) in deionized water (50 mL) was added at 10–12 °C, resulting mixture was stirred at room temperature overnight. Reaction mixture was washed with CH_2Cl_2 (2 × 25 mL) then acidified to pH 2 with 6 M HCl. Product was extracted with CH_2Cl_2 (2 × 50 mL and 1 × 25 mL), washed with water (1 × 50 mL) and brine (1 × 25 mL), dried with anhydrous Na_2SO_4, filtered through a short plug of silica gel. Solvent was evaporated under reduced pressure to afford compound **1** (8.46 g, 86.7%) as light yellow oil.

S-(6-chloro-6-oxohexyl) ethanethioate (2)

To a solution of compound **1** (5 g, 26.4 mmol) and DMF (0.1 mL, 1.31 mmol) in dichloromethane (150 mL), under argon atmosphere, oxalyl chloride (3.4 mL, 39.4 mmol) was added dropwise at 0–3 °C, then stirred at room temperature for 2 hours. Reaction mixture was evaporated to dryness. Obtained intermediate **2** was used immediately in the next step.

S-(6-((2-(1H-imidazol-4-yl)ethyl)amino)-6-oxohexyl) ethanethioate (3)

To a suspension of histamine dihydrochloride (9.67 g, 52.6 mmol) in DMF (52 mL), triethylamine (29.5 mL, 210 mmol) was added under argon atmosphere, than solution of intermediate **2** (all from previous step, 26.4 mmol) in DMF (26 mL) was added dropwise at 0–5 °C, mixture was stirred at the same temperature for 1 hour, then at room temperature overnight. Reaction mixture was diluted with deionized water (150 mL), product was extracted with CH_2Cl_2 (1 × 200 mL) and a mixture of CH_2Cl_2 with MeOH (2 × 100 mL CH_2Cl_2 : 25 mL MeOH). Organic layers were combined, evaporated to dryness and subjected to flash chromatography (CH_2Cl_2/MeOH 90:10) to afford compound **3** (5.42 g, 72.8 %) as white crystals, m.p 114–117 °C, R_f 0.7 (4:1 CH_2Cl_2/MeOH).

^{1}H NMR (400 MHz, DMSO-d_6) δ 11.70 (brs, 1H) 7.83 (s, 1H), 7.52 (s, 1H), 6.78 (s, 1H), 3.23–3.28 (m, 2H), 2.82 (t, J = 7.2 Hz, 2H), 2.62 (t, J = 7.4 Hz, 2H), 2.32 (s, 3H), 2.04 (t, J = 7.4 Hz, 2H), 1.45–

1.53 (m, 4H), 1.23–1.32 (m, 2H). ^{13}C NMR (101 MHz, DMSO-d_6) δ 195.31, 171.80, 134.57, 38.64, 35.21, 30.57, 28.91, 28.26, 27.74, 24.75.

N-(2-(1H-imidazol-4-yl)ethyl)-6-mercaptohexanamide (IMHA)

To a solution of compound **3** (3.5 g, 12.4 mmol) in MeOH (40 mL), under argon atmosphere, NaOMe (9.1 mL, 49.4 mmol, 30% w/w in MeOH) was added dropwise at room temperature, and was allowed to stir overnight. Reaction mixture was cooled in an ice bath, neutralized (~7 pH) with 2 M HCl and brine (10 mL) was added. Product was extracted with CH_2Cl_2 (1 × 100 mL) and a mixture of CH_2Cl_2 with MeOH (50 mL CH_2Cl_2 : 10 mL MeOH). Organic layers were combined and evaporated to dryness. Remaining mixture was dissolved in a mixture of CH_2Cl_2 with MeOH (20 mL CH_2Cl_2 : 5 mL MeOH), DTT (3.8 g, 24.7 mmol) then triethylamine (3.44 mL, 24.7 mmol) was added, and allowed to stir under argon atmosphere overnight. Solvents were evaporated to dryness and subjected to flash chromatography (CH_2Cl_2/MeOH 85:15) to give **IMHA** (1.47 g, 49%) as white crystals, m.p 130–131 °C, R_f 0.65 (4:1 CH_2Cl_2/MeOH).

^{1}H NMR (400 MHz, DMSO-d_6) δ 7.85 (t, J = 5.7 Hz, 1H), 7.52 (d, J = 1.1 Hz, 1H), 6.77 (d, J = 1.1 Hz, 1H), 3.24 (td, J = 7.5, 5.5 Hz, 2H), 2.60 (t, J = 7.4 Hz, 2H), 2.45 (q, J = 7.3 Hz, 2H), 2.21 (t, J = 7.7 Hz, 1H), 2.03 (t, J = 7.3 Hz, 2H), 1.43–1.55 (m, 4H), 1.25–1.33 (m, 2H). ^{13}C NMR (101 MHz, DMSO-d_6) δ 171.88, 134.57, 134.32, 116.89, 38.64, 35.31, 33.12, 27.36, 27.00, 24.74, 23.65.

S-(6-(methylamino)-6-oxohexyl) ethanethioate (4)

To a solution of compound **1** (4.755 g, 25 mmol) in dichloromethane (125 mL), DMAP (0.305 g, 2.5 mmol) and EDCI Cl (6.47 g, 33.75 mmol) was added at 0–5 °C under argon atmosphere. Then methylamine (40% solution in MeOH, 3.82 mL, 37.5 mmol) was added dropwise followed by addition of triethylamine (5.23 mL, 37.5 mmol) at 0–5 °C and left to stir at room temperature for 24 hours. Reaction mixture was washed with water (1 × 20 mL), 1 M HCl (2 × 25 mL), saturated $NaHCO_3$ with brine (1 × 50 mL, 1:1) and dried with anhydrous Na_2SO_4. Solvent was evaporated to dryness and subjected to flash chromatography (CH_2Cl_2/MeOH 96:4) to give product **4** (3.86 g, 76% yield, containing 5% of compound **5**) as white crystals, R_f 0.35.

^{1}H NMR (400 MHz, $CDCl_3$) δ 5.83 (s, 1H), 2.83 (t, *J* = 7.3 Hz, 2H), 2.78 (d, *J* = 3.8 Hz, 3H), 2.30 (s, 3H), 2.17 (t, *J* = 7.6 Hz, 2H), 1.67–1.53 (m, 4H), 1.41–1.35 (m, 2H). ^{13}C NMR (101 MHz, $CDCl_3$) δ 196.14, 173.74, 36.34, 30.74, 29.34, 28.94, 28.41, 26.42, 25.27.

6-mercapto-N-methylhexanamide (5) (Fragment molecule)

To a solution of compound **4** (3 g, 14.78 mmol) in MeOH (40 mL), under argon atmosphere, NaOMe (2.8 mL, 14.78 mmol, 30% w/w in MeOH) was added dropwise at room temperature, and was allowed to stir for 2 hours. Solvent was evaporated and remaining mixture was dissolved in water (20 mL) and acidified with 3 M HCl (5 mL). Product was extracted with CH_2Cl_2 (3 × 30 mL), washed with brine (1 × 10 mL), dried with anhydrous Na_2SO_4. Solvent was evaporated to dryness under reduced pressure. Remaining mixture was dissolved in CH_2Cl_2 (20 mL), DTT (1.13 g, 7.38 mmol) then triethylamine (1.03 mL, 7.38 mmol) was added, and allowed to stir under argon atmosphere overnight. Reaction mixture was washed with 0.5 M HCl (1 × 10 mL), 0.25 M HCl (2 × 20 mL), water (1 × 20 mL), dried with anhydrous Na_2SO_4. Solvent was evaporated to dryness and subjected to flash chromatography (CH_2Cl_2/MeOH 97:3) to give product **5** (1.88 g, 79%) as white crystals, m.p 30–31 °C, R_f 0.36 (96:4 CH_2Cl_2/MeOH).

^{1}H NMR (400 MHz, DMSO-d_6) δ 7.68 (s, 1H), 2.54 (d, *J* = 4.6 Hz, 3H), 2.45 (q, *J* = 7.3 Hz, 2H), 2.20 (t, *J* = 7.6 Hz, 1H), 2.03 (t, *J* = 7.4 Hz, 2H), 1.55–1.43 (m, 3H), 1.33–1.26 (m, 2H). ^{13}C NMR (101 MHz, DMSO-d_6) δ 172.41, 35.21, 33.10, 27.42, 25.38, 24.71, 23.63.

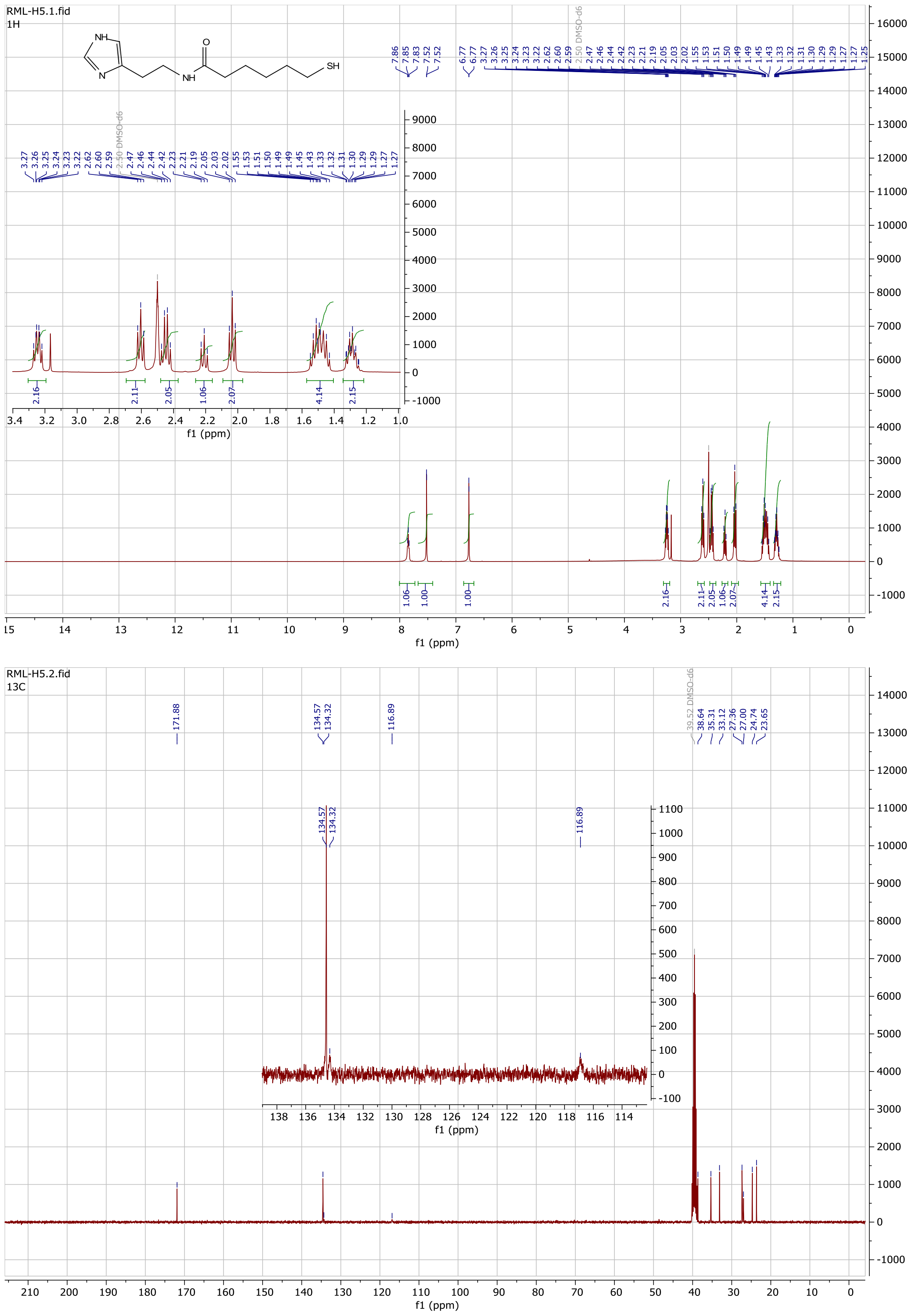

Figure S1. ^{1}H NMR and ^{13}C NMR spectra of N-(2-(1H-imidazol-4-yl)ethyl)-6-mercaptohexanamide (IMHA).

Results

Assignments of Raman Bands

Figure S2 compares Raman spectra of powder IMHA, fragment molecule, and histidine and the spectra of fragment molecule dissolved in H_2O and D_2O. Figure S2A compares spectra of Im-molecules (His and IMHA) with a spectrum of fragment molecule, which allows to identify Raman bands related with Im ring vibrations. Spectra in Figure S2B of powder fragment molecule and dissolved in H_2O and D_2O allow to ascertain the amide and thiol groups related modes. Table 1 presents the spectral band assignment of IMHA based on DFT calculations, temperature-Raman, H_2O/D_2O exchange Raman measurements, and the literature data of similar compounds [1–6].

S–H stretching vibration appears as an intense mode at 2572 cm^{-1}, while corresponding S–D band visible at 1876 cm^{-1} in D_2O solution Raman spectrum of fragment molecule (Figure S2B). Alkanethiol-universal C–S stretching duplet of $-CH_2-CH_2-S-$ moiety in gauche/trans conformations, which is typically found in 600–750 cm^{-1} region, is less straightforward to identify, because the same spectral region is occupied by rocking vibrations of five-methylene-segment and Im deformations. The rocking motion is highly sensitive to the packing of carbohydrate chains [7], thus the initially sharp and separated bands become broadened with the melting of the IMHA. The 653 cm^{-1} mode appears as medium intensity feature in IMHA and L-histidine spectra and as a very weak mode in fragment molecule spectrum in Figure S2A. We tentatively assigned it to Im ring motion with some character expected from the stretching of S–C in gauche conformation. This band becomes greatly intensified with the fragment compound dissolution in H_2O (Figure S2B) suggesting that C–S bond undergoes isomerization reaction from gauche to trans. Notably, in D_2O solvent this band is less intense and upshifted by 5 cm^{-1} due to the decoupling of vibrational modes. Such a frequency upshift induced by D_2O/H_2O exchange was observed previously for the $\nu(C-S)_G$ mode of cysteamine cation ($SH-CH_2-CH_2-NH_3^+$) [8]. Similarly, in temperature-Raman measurements the mode at 654 cm^{-1} intensify when IMHA is heated to a melting point. Thus, we conclude the 653 cm^{-1} band's assignment to the Im motion + $\nu(C-S)_G$. Based on H_2O/D_2O exchange and temperature-Raman measurements, modes at 732 and 753 cm^{-1} are assigned to methylene rocking, while 711 cm^{-1} mode to $\nu(C-S)_T$. DFT modeling predicts $\nu(C-S)_T$ at 712 cm^{-1} (potential energy distribution, PED = 50%).

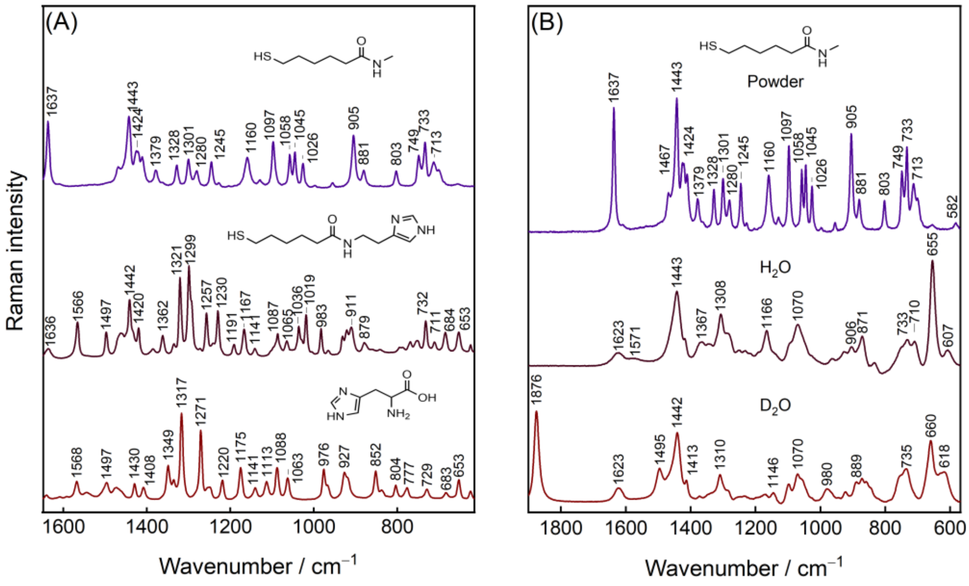

Figure S2. (A) Raman spectra of powder IMHA, fragment molecule, and histidine. (B) Raman spectra of powder fragment molecule and the 0.33 M solutions in H_2O and D_2O.

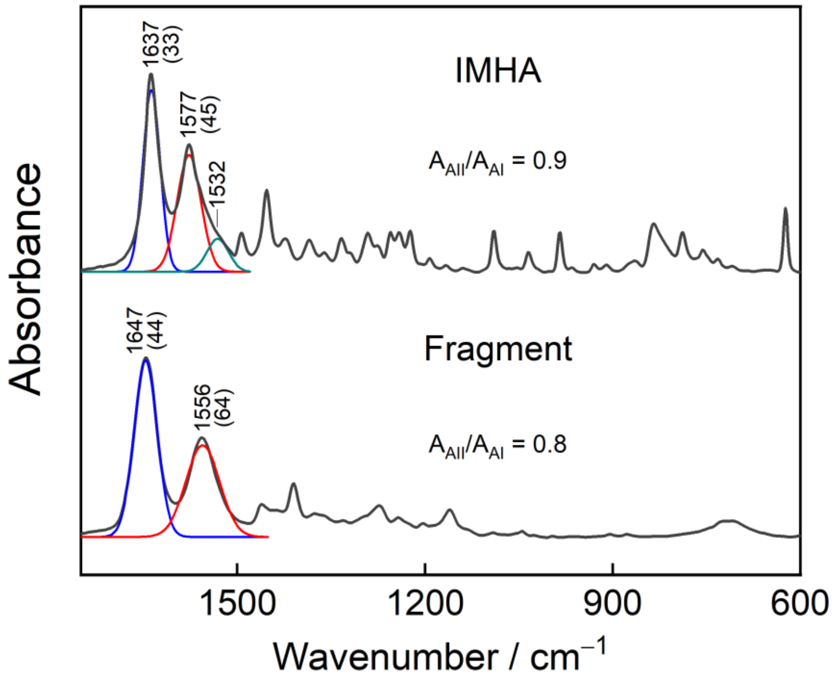

Figure S3. FTIR transmission spectra of IMHA and fragment molecule powders dispersed in KBr pellets. Am-I and Am-II spectral modes were approximated using Gaussian shape components. The full-widths at half maxima of Am-I and Am-II bands are given next to corresponding wavenumbers.

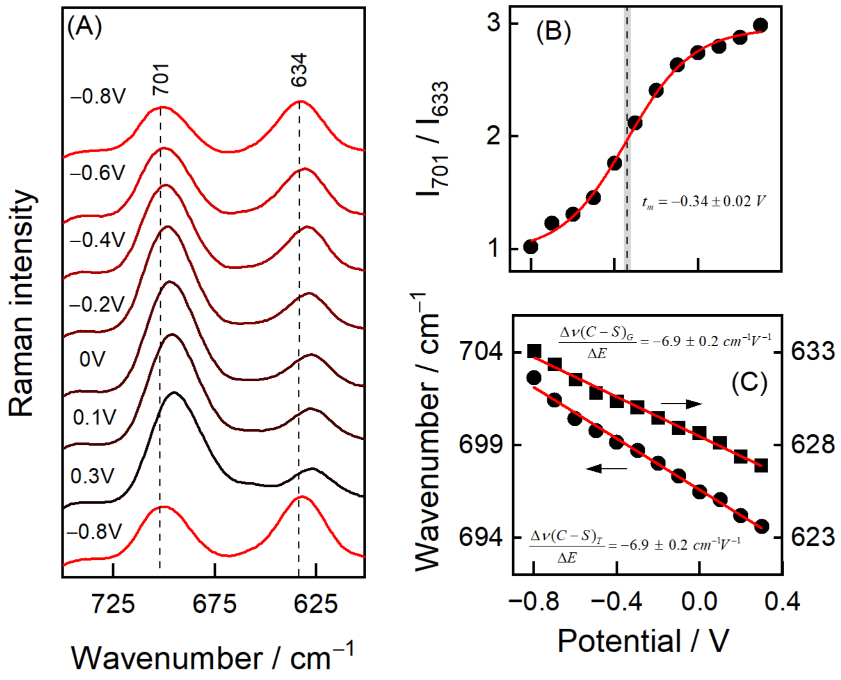

Figure S4. (A) SHINERS spectra of IMHA adsorbed on smooth Au electrode at indicated potentials in the 600–750 cm^{-1} spectral region. Spectra were recorded in 0.1 M Na_2SO_4 aqueous solution containing 0.01 M phosphate buffer (pH 7). The excitation wavelength was 785 nm. (B) Dependence of relative integrated intensity I_{701}/I_{633} ratio of IMHA bands on electrode potential fitted with the sigmoidal curve (Boltzmann model, R^2= 0.99658). The transition midpoint potential value was determined at –0.34 ± 0.02 V, shown by the dashed line. The grey bar around the midpoint line shows the error in the fitting value. (C) Dependence of ν(C–S)$_G$ and ν(C–S)$_T$ modes wavenumbers of IMHA on electrode potential. Data in **(**B**)** and **(C)** sections were presented as an average of three independent measurements.

References

1. Ashikawa, I.; Itoh, K. Raman spectra of polypeptides containing L-histidine residues and tautomerism of imidazole side chain. *Biopolymers* **1979**, *18*, 1859–1876. https://doi.org/10.1002/bip.1979.360180804.
2. Matulaitienė, I.; Kuodis, Z.; Eicher-Lorka, O.; Niaura, G. SERS characterization of imidazole ring terminated self-assembled monolayer formed from lipoic acid histamide on silver electrode. *J. Electroanal. Chem*. **2013**, *700*, 77–85. https://doi.org/10.1016/j.jelechem.2013.04.017.
3. Mesu, J.G.; Visser, T.; Soulimani, F.; Weckhuysen, B.M. Infrared and Raman spectroscopic study of pH-induced structural changes of L-histidine in aqueous environment. *Vib. Spectrosc*. **2005**, *39*, 114–125. https://doi.org/10.1016/j.vibspec.2005.01.003.
4. Matulaitienė, I.; Pociūtė, E.; Kuodis, Z.; Eicher-Lorka, O.; Niaura, G. Interaction of 4-imidazolemethanol with a copper electrode revealed by isotope-edited SERS and theoretical modeling. *Phys. Chem. Chem. Phys*. **2015**, *17*, 16483–16493. https://doi.org/10.1039/c5cp01290b.
5. Toyama, A.; Ono, K.; Hashimoto, S.; Takeuchi, H. Raman spectra and normal coordinate analysis of the N1-H and N3-H tautomers of 4-methylimidazole: Vibrational modes of histidine tautomer markers. *J. Phys. Chem. A*. **2002**, *106*, 3403–3412. https://doi.org/10.1021/jp0124185
6. Niaura, G. **2014**. "Raman Spectroscopy in Analysis of Biomolecules." In Encyclopedia of Analytical Chemistry, 1–34. John Wiley & Sons, Ltd. https://doi.org/10.1002/9780470027318.a0212.
7. Nelson, P.N. Chain Length and Thermal Sensitivity of the Infrared Spectra of a Homologous Series of Anhydrous Silver(I) n –Alkanoates. *Int. J. Spectrosc*. **2016**, *2016*, 1–9. https://doi.org/10.1155/2016/3068430.
8. Riauba, L.; Niaura, G.; Eicher-Lorka, O.; Butkus, E. A study of cysteamine ionization in solution by Raman spectroscopy and theoretical modeling. *J. Phys. Chem. A*. **2006**, *110*, 13394–13404. https://doi.org/10.1021/jp063816g.

*these references are also cited in the Manuscript.
